# Predicting psychotic-like experiences among adolescents: the interplay of childhood trauma, cognitive biases, neuroticism, and depression

**DOI:** 10.1186/s13034-025-00878-5

**Published:** 2025-03-14

**Authors:** Chang Xi, Xin Xu, Song Wang

**Affiliations:** 1 Department of Psychology, Hunan University of Chinese Medicine, Changsha, 410208 Hunan China; 2 Department of Psychology, School of Education, Soochow University, Suzhou, China; 3 College of Information and Intelligence, Hunan Agricultural University, Changsha, China

## Abstract

**Background:**

Childhood trauma, cognitive biases, neuroticism, and depression have emerged as crucial risk markers for psychotic-like experiences (PLEs). However, the interplay among these variables in influencing the risk of PLEs remains largely unexplored. This study aims to investigate the effect of the complex relationship between childhood trauma, cognitive biases, neuroticism, and depression on the risk of PLEs among adolescents.

**Methods:**

A total of 4,087 adolescents from three senior high schools were recruited for this study. We utilized the Community Assessment of Psychic Experiences to measure PLEs, the Childhood Trauma Questionnaire to assess childhood trauma, the Davos Assessment of Cognitive Biases scale to evaluate cognitive biases, the neuroticism subscale of the Neuroticism-Extraversion-Openness Personality Inventory to assess neuroticism, and the Patient Health Questionnaire to measure depression. Psychiatric diagnoses were screened using a self-report questionnaire.

**Results:**

Childhood trauma, cognitive biases, neuroticism and depression were all associated with an increased risk of PLEs. Participants who had experienced childhood trauma in conjunction with depression, cognitive biases, or neuroticism exhibited a significantly higher risk of endorsing PLEs compared to those who had solely encountered childhood trauma. Path analysis revealed that cognitive biases, neuroticism and depression are significant mediators of the relationship between childhood trauma and PLEs. The model explained 44.7% of the variance in PLEs.

**Conclusions:**

Our study highlights cognitive biases, neuroticism, and depression as key mediators linking childhood trauma to PLEs, recognizing the complex interplay among these factors is crucial and should be integrated into clinical screening and therapeutic strategies to mitigate the risk of PLEs.

**Supplementary Information:**

The online version contains supplementary material available at 10.1186/s13034-025-00878-5.

## Introduction

Psychotic-like experiences (PLEs) refer to subthreshold psychotic symptoms that are associated with an increased risk of transitioning to psychotic disorders [1, 2]. PLEs are relatively common, particularly in adolescents [3]. Studies have reported a median prevalence of 17% among children aged 9–12 years and 7.5% among adolescents aged 13–18 years across various countries, including Australia, Spain, and Japan [3]. In China, a relatively higher prevalence of 20.7% has been identified among individuals aged 10 to 23.3 years, as compared to other countries [4]. A substantial body of research indicates that PLEs can lead to the development of psychotic disorders [5], non-psychotic disorders [6, 7], as well as an elevated risk of various adverse outcomes, encompassing emotional disturbances, suicidal tendencies and social dysfunction [8–10]. Therefore, identifying the influencing factors of PLEs in adolescents is crucial for the early prevention of mental health issues.

PLEs share several risk factors with psychotic disorders, including childhood trauma, socioeconomic status, place of residence, cognitive dysfunctions, depressive symptoms, and individual personality traits [11–15]. Accumulating evidence suggests that individuals with a history of childhood trauma are more likely to experience PLEs [16], with trauma considered a significant factor in subclinical psychotic symptoms [17, 18]. Nonetheless, exposure to traumatic life events alone is insufficient to account for the development of psychosis [19]. Recent studies have proposed that traumatic life events increase the risk of psychosis through cognitive biases, which refer to systematic errors in cognitive processing and irrational judgment [20]. Cognitive biases, supported by the cognitive model of psychosis, may play a critical role in the formation and persistence of psychotic symptoms [21]. According to this model, vulnerable predispositions (e.g., certain personality traits) interact with traumatic life events and adverse environments, leading to emotional changes and cognitive biases that contribute to the development of subthreshold or threshold positive psychotic symptoms, including PLEs [14, 19, 22].

Among personality traits linked to the risk of mental disorders, neuroticism is particularly noteworthy. Neuroticism is elevated in a wide range of psychological disorders, including schizophrenia-spectrum disorders, major depression, and generalized anxiety [23–25]. It is characterized by heightened emotional reactivity, specifically a tendency to become easily aroused and to slowly return to emotional baseline [26, 27]. Individuals with high levels of neuroticism are more prone to negative emotions, such as sadness, fear, and anger. Increased neuroticism has been associated with the persistence of early PLEs [15].

Depressive symptoms are also considered significant predictors of PLEs [14]. Depression is highly prevalent among individuals with psychosis, as well as among those at ultra-high risk of developing psychosis [28, 29]. Previous studies have shown that individuals with mood disorders face a fourfold increased risk of experiencing psychotic symptoms [30]. Furthermore, depressive symptoms are associated with an increased likelihood of PLEs in the general population [31, 32].

In summary, PLEs are likely to result from the interplay between childhood trauma and other processes involved in the development of psychosis. Specifically, the combination of childhood trauma, cognitive biases, neuroticism, and depression appears to contribute to the emergence of PLEs. Despite the importance of this topic, relatively few studies have examined the mechanisms by which these factors interact to impact PLEs. The current study aims to investigate how childhood trauma, cognitive biases, neuroticism, and depression jointly influence the risk of PLEs in adolescents. Based on the cognitive model of psychosis and prior research, we hypothesize that childhood trauma increases the risk of PLEs through dysfunctional cognitive processes (cognitive biases), emotional changes (depression), and personality traits (neuroticism). Specifically, we propose that childhood trauma alters cognitive processing through neuroticism, which in turn increases the frequency of PLEs, and that childhood trauma also influences mood, leading to depressive symptoms and a heightened risk of PLEs.

## Methods

### Participants

A convenience sample of adolescents was recruited from three senior high schools in Hunan Province, China. Participants with a history of psychiatric disorders or neurological conditions, based on self-reported data, were excluded from the analysis (98 participants excluded). The final sample included 4,087 participants (2,026 females, 2,061 males), aged 14–19 years (M = 16.16, SD = 0.88). This study was approved by the Institutional Review Board (IRB) of the First Affiliated Hospital of Hunan University of Chinese Medicine, and written informed consent was obtained from all participants.

## Assessments

### Demographic data

A sociodemographic and clinical questionnaire was designed to collect basic information, including age, sex, residence, only-child status, economic status, and parental marital status. Economic status was measured using the MacArthur Scale [33], a 10-rung ladder that assesses perceived socioeconomic status, with the top rung representing the highest socioeconomic status and the bottom representing the lowest. This scale has been validated among Chinese adults [34].

## Psychotic-like experiences (PLEs)

PLEs were measured using the Community Assessment of Psychic Experiences (CAPE-P15) [35]. The CAPE-P15 consists of two subscales assessing the frequency and distress of psychotic-like experiences across 15 items. These items are categorized into three factors: persecutory ideation (PI), bizarre experiences (BE), and perceptual abnormalities (PA). A mean cut-off score of 1.47 on both subscales (frequency and distress) was used to screen for ultra-high risk of psychosis [36]. The Chinese version of the CAPE-P15 has demonstrated satisfactory psychometric properties among adolescents [37], with a Cronbach’s α of 0.896 in this study.

## Depression

Depressive symptoms were assessed using the Patient Health Questionnaire-9 (PHQ-9), a widely used tool for screening and evaluating the severity of depression [38]. The PHQ-9 consists of nine items rated on a scale of 0 to 3, with total scores ranging from 0 to 27. In this study, a cut-off score of 15 was adopted to identify positive cases. The Chinese version of the PHQ-9 has demonstrated good psychometric properties [39], with a Cronbach’s α of 0.891.

### Neuroticism

Neuroticism was measured using the neuroticism subscale of the Neuroticism-Extraversion-Openness Personality Inventory (NEO-PI). This subscale includes 12 items rated on a 5-point Likert scale, with scores ranging from 5 to 60. Higher scores indicate higher levels of neuroticism. The subscale includes six facets: anxiety, angry-hostility, depression, self-consciousness, impulsiveness, and vulnerability. The NEO-PI has been validated for use in Chinese populations [40], and the Cronbach’s α for the neuroticism subscale in this study was 0.799.

## Cognitive biases

Cognitive biases were assessed using the Davos Assessment of Cognitive Biases Scale (DACOBS) [41]. The DACOBS contains 42 items rated on a 7-point Likert scale, with higher scores indicating more pronounced cognitive biases. The scale includes seven subscales: jumping to conclusions, belief inflexibility, attention to threat bias, external attribution bias, social cognition problems, subjective cognitive problems, and safety behaviors. The Cronbach’s α for the DACOBS was 0.921 in this sample.

## Childhood trauma

To measure childhood trauma experience before their 16 years old, we the short form of the Childhood Trauma Questionnaire (CTQ-SF) [42]. The CTQ-SF is a 28-item questionnaire using a seven-point Likert scale (1–5). Higher total scores indicate more childhood trauma experienced. Of the 28 items, 25 items are included in 5 subscales (emotional abuse, physical abuse, sexual abuse, emotional neglect, and physical neglect). The 3 remaining items are used to test the validity of each questionnaire. This scale is considered to be a sensitive and valid screening questionnaire for childhood trauma. Four subscales (emotional abuse, physical abuse, emotional neglect, and physical neglect) were used in this study, with a Cronbach’s α of 0.697.

### Data analysis

All analyses were conducted with SPSS 26.0 and the AMOS 26.0. First, we conducted a series of univariate logistic regression analyses to investigate whether the variables made an independent contribution to PLE. We adopted a mean score cut-off for ultra-high risk for psychosis of 1.47 for both frequency and distress scores on CAPE-P15 [36]. Our variables were: Depression, cognitive biases, neuroticism and childhood trauma. With regard to cognitive biases and neuroticism, we divided participants into two groups using a cut-off of 2 S.D. from the mean. Those who scored at least 195.75 points (with 294 as the highest) on the DACOBS were classified as endorsing cognitive biases, and who scored at least 51.13 points (with 60 as the highest) on the neuroticism subscale of the NEO-PI were classified as showing neuroticism. To control for potential confounders, we included age, gender, residence, single child status, economic status and parental marital status in regression models. Then multivariate logistic regressions were carried out using all predictors included in univariate analyses. We also performed stratified logistic regression analysis to identify variables that affect the association between these variables and PLE in different subgroups.

In the next step, we performed confirmatory factor analyses (CFA) of the latent variables. In cases where the original structure of the variables did not reach satisfactory model fit, we modified them by excluding unsatisfactory items. Path analysis was conducted with continuous variables, and for the outcome variable we used the CAPE-P15 frequency score. We also examined the potential multicollinearity of all variables using the variance inflation factor (VIF) [43] indicator. Goodness of model fit was verified following guidelines from literature [44]: RMSEA < 0.06 (The Root Mean Square Error of Approximation); SRMR < 0.08 (The Standardized Root Mean Square Residual); CFI ≥ 0.90 (Confirmatory Fit Index) and GFI ≥ 0.90 (Goodness-of-Fit).

## Results

### Characteristics of the sample

Table 1 presents sample characteristics.

**Table 1:** Sample characteristics

Variables	Mean ± SD or *n* (%)
Gender	
Male	2061 (50.4%)
Female	2026 (49.6%)
Age	16.16 (0.88)
Residence	
Urban	2610 (63.9%)
Town	694 (17.0%)
Rural	783 (19.2%)
Single child status	
Yes	370 (9.1%)
No	3717 (90.9%)
Economic status (total score)	5.45 (1.42)
Parental marital status	
Married	3476 (85.1%)
^a^Not current married	611 (14.9%)
PHQ-9 (total score)	6.18 (5.20)
Participants with depression	306 (7.5%)
Participants without depression	3781 (92.5%)
CTQ (total score)	35.42 (10.12)
Participants with Childhood trauma	2121 (51.9%)
Participants without Childhood trauma	1966 (48.1%)
DACOBS (total score)	125.55 (35.10)
Neuroticism subscale of the NEO-PI (total score)	33.05 (9.04)
Frequency subscale of CAPE-P15 (total score)	1.36 (0.41)

PHQ-9, 9-item Patient Health Questionnaire; CTQ, Childhood Trauma Questionnaire; DACOBS, Davos Assessment of the Cognitive Biases Scale; NEO-PI, NEO Personality Inventory Revised; CAPE-P15, 15-item positive subscale of the community assessment of psychic experiences; ^a^Not current married included separated, divorced, and widowed

### Logistic regression analyses

Univariate regression models showed that all predictors were statistically significant. People with higher level of neuroticism had the highest risk of PLEs (OR 14.61, 95%CI 8.94–23.90, *p* < 0.001). Depression also significantly increased the likelihood of PLEs (OR 12.06, 95%CI 9.23–15.76, *p* < 0.001), as well as childhood trauma (OR 3.08, 95%CI 2.59–3.65, *p* < 0.001) and cognitive biases (OR 6.36, 95%CI 4.16–9.74, *p* < 0.001). Multivariate regression analyses showed that all predictors remained significant in the model for PLEs (all *p*-values < 0.001). Detailed results are presented in Table 2.

**Table 2:** OR from univariate and multivariate logistic regressions predicting psychotic-like experiences

	Yes n (%)	No n (%)	Univariate		Multivariate	
			OR (95% CI)	*p* value	OR (95% CI)	*p* value
Psychotic-like experiences	842 (20.6%)	3245 (79.4%)				
Depression	221 (26.2%)	621 ( (73.8%)	12.06 (9.23–15.76)	< 0.001	7.45 (5.60–9.92)	< 0.001
Cognitive biases	58 (6.9%)	784 (93.1%)	6.36 (4.16–9.74)	< 0.001	2.88 (1.71–4.83)	< 0.001
Neuroticism	81 (9.6%)	761 (90.4%)	14.61 (8.94–23.90)	< 0.001	4.91 (2.79–8.64)	< 0.001
Childhood trauma	622 (73.9%)	220 (26.1%)	3.08 (2.59–3.65)	< 0.001	2.56 (2.14–3.06)	< 0.001

OR, odds ratio; CI, 95% confidence interval. Adjusted for age, gender, residence, single child status, economic status and parental marital status

Stratified logistic regression analysis were further performed to identify variables that may modify the association between predictors and PLEs (Table 3). Results showed that, compared to individuals who solely endured childhood trauma, the risk of PLEs increased significantly among those with childhood trauma when it was combined with neuroticism (OR 8.94, 95% CI 5.23–15.27, *p* < 0.001), depression (OR 9.02, 95%CI 6.63–12.27, *p* < 0.001) or cognitive biases (OR 4.86, 95%CI 2.91–8.13, *p* < 0.001).

**Table 3:** Interaction effects between childhood trauma, cognitive biases, neuroticism and depression

			No (OR, 95% CI)	Yes (OR, 95% CI)	*p* value
			*Childhood trauma*		
Depression	Yes (n)	306	Reference	1.91 (1.01–3.60)	< 0.05
Cognitive biases	Yes (n)	96	Reference	1.57 (0.58–4.24)	0.371
Neuroticism	Yes (n)	102	Reference	0.84 (0.20–3.51)	0.808
			*Neuroticism*		
Childhood trauma	Yes (n)	2121	Reference	8.94 (5.23–15.27)	< 0.001
Cognitive biases	Yes (n)	96	Reference	6.99 (1.40-34.82)	< 0.05
Depression	Yes (n)	306	Reference	1.98 (0.99–3.98)	0.053
			*Depression*		
Childhood trauma	Yes (n)	2121	Reference	9.02 (6.63–12.27)	< 0.001
Cognitive biases	Yes (n)	96	Reference	7.90 (2.67–23.37)	< 0.001
Neuroticism	Yes (n)	102	Reference	2.05 (0.67–6.32)	0.211
			*Cognitive biases*		
Childhood trauma	Yes (n)	2121	Reference	4.86 (2.91–8.13)	< 0.001
Depression	Yes (n)	306	Reference	2.29 (0.96–5.43)	0.061
Neuroticism	Yes (n)	102	Reference	4.46 (0.74–26.83)	0.103

OR, odds ratio; CI, 95% confidence interval. Adjusted for age, gender, residence, single child status, economic status and parental marital status

### Path analyses

**Fig. 1:**
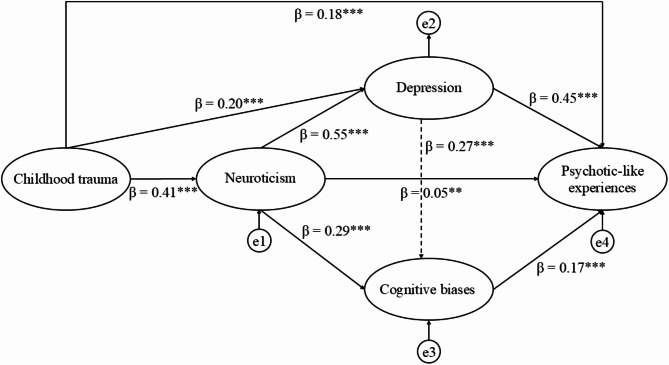
Model of the relationship between childhood trauma and psychotic-like experiences with neuroticism, depression, and cognitive biases as mediators. β = standardized regression coefficients, e = residual errors, **p < 0.01, ***p < 0.001

Confirmatory factor analyses showed a good fit for all scales except for DACOBS and Neuroticism, thus we excluded six low-loading items (1, 12, 25, 26, 27, 30) of DACOBS and three low-loading items (1, 4, 7) of Neuroticism to reach satisfactory model fit (Supplementary Table S1). Then we tested the hypothesized model to examine associations among childhood trauma, neuroticism, depression, cognitive biases and PLEs. There was no sign of multicollinearity as indicated by the VIF of all predictors < 1.5. The pathway linking childhood trauma to cognitive biases was found to be insignificant and was therefore excluded to achieve the most parsimonious model with a good fit. The final model explained 44.7% of the variance in PLEs. All of the model fit indices were satisfactory: RMSEA = 0.017 (90% CI 0.000–0.049), CFI = 1.000, SRMR = 0.005, GFI = 1.000. The model with standardized path coefficients is presented in Fig. 1.

Childhood trauma had a significant standardized indirect effect on PLEs through all the other variables (β = 0.251, 95% CI 0.231–0.269, *p* < 0.05). To find out if the mediation involving cognitive biases, PLEs, and depression was full or partial, we checked for a standardized direct effect of childhood traumatic life events on PLEs. This standardized direct effect was significant (β = 0.177, 95% CI 0.147–0.209, *p* < 0.05), meaning that the mediation by the other variables was only partial. The standardized total effect of childhood trauma on PLEs was also statistically significant (β = 0.428, 95% CI 0.400–0.454, *p* < 0.05).

The bootstrapping analysis showed that depression had the biggest impact on PLEs, with a standardized total effect size of 0.490 (comprising a direct effect of 0.445 and an indirect effect of 0.045). Childhood trauma was the next biggest factor, with a standardized total effect size of 0.428 (consisting of a direct effect of 0.177 and an indirect effect of 0.251). Neuroticism also played an important role, with a standardized total effect size of 0.364, which was mostly indirect (0.318) with a small direct effect (0.045). Cognitive biases had a total effect size of 0.166, which was entirely direct. All these variables had a significant overall effect on PLEs. A detailed breakdown of each factor’s effect size was shown in Table 4.

**Table 4:** Effect analysis of related influencing factors for psychotic-like experiences in adolescents. (*n* = 4087)

Variables	Direct effect	Indirect effect	Total effect
Depression	0.445	0.045	0.490
Cognitive biases	0.166	0.000	0.166
Neuroticism	0.045	0.318	0.364
Childhood trauma	0.177	0.251	0.428

## Discussion

In the current study, we reported a prevalence rate of 20.6% for PLE among adolescents, which is consistent with one prior study conducted in China [4]. And we further confirmed that the risk of PLEs results from the interplay between childhood trauma, cognitive biases, neuroticism and depression among adolescents. In line with previous clinical and non-clinical studies [18, 45, 46], our findings suggest a pathway from childhood traumatic life events to psychosis proneness. For the first time, our findings revealed that the paths from childhood trauma to PLEs among adolescents go through important, but partial mediators– cognitive biases, neuroticism and depression, it is consistent with the cognitive model of psychosis.

Although previous studies confirmed the role of childhood trauma, cognitive biases, neuroticism and depression in PLEs, this study considered these predictors in combination for the first time. The non-adjusted odds ratio (OR) for the association between the markers under consideration and PLEs decreased in the multivariate logistic regression, after controlling for all predictors included in the analysis and adjusting for age, gender, residence, single-child status, economic status, and parental marital status. However, childhood trauma, cognitive biases, neuroticism, and depression remained significant influencing factors of PLEs. Therefore, our findings suggest that these markers each contribute meaningfully and independently to PLEs in the non-clinical adolescent population.

Even though multivariate logistic regression provided results suggesting an independent contribution of these markers to PLEs, it did not shed light on their potential interactive effect. Our subsequent analysis of interaction between these markers revealed that the combination of childhood trauma with neuroticism, depression, or cognitive bias had a stronger impact on PLEs than childhood trauma alone. For instance, among people who had experienced childhood trauma combined with depression or neuroticism, the risk of PLEs was increased by approximately nine times compared to those who experienced only childhood trauma. Similarly, for individuals with childhood trauma and cognitive biases, the risk of PLEs was approximately 4.86 times higher compared to those without cognitive biases. These findings strongly suggests that these important markers should be considered in combination during screening for risk of PLEs.

Despite progress in researching the mechanisms responsible for PLEs, little is known about how these potential markers might interact in impact PLEs. This study aims to provide a theoretical model that recognizes the complexity of PLEs, extending existing knowledge by demonstrating that the risk of PLEs is largely explained (44.7% of the variance) by the interplay between childhood trauma, cognitive biases, neuroticism, and depression. These findings are consistent with prior research highlighting the interconnectedness of environmental risks (e.g., exposure to traumatic life events), cognitive biases, and depression, all of which have been linked to the risk of psychosis and PLEs [45, 47–49]. The cognitive model of psychosis posits that exposure to traumatic events alters cognitive architecture, leading to dysfunctional responses to environmental stressors [50]. Recent studies suggest that cognitive biases significantly mediate the relationship between traumatic life events and psychosis risk in both clinical and non-clinical populations [45, 46]. Additionally, personality traits such as neuroticism are associated with psychosis proneness in both the general and clinical high-risk populations [51–54]. Consequently, this study supports the view that a combination of childhood trauma, neuroticism, cognitive biases, and depression provides a robust influential framework for PLEs and may be of particular interest for clinical screening and future research.

Actually, most individuals who suffer from PLEs achieve remission, with only approximately 7% of those with PLEs developing into full-blown psychosis [48]. Identifying high-risk factors associated with PLEs is crucial for detecting the risk of psychosis and preventing the onset of mental disorders. Our findings indicate that considering the intricate interplay between childhood trauma, neuroticism, cognitive biases, and depression could enhance the effectiveness of psychosis screening procedures, where a comprehensive assessment of all markers is essential. Regarding the application of our results to therapeutic interventions, it is noteworthy that cognitive biases and depression have already been successfully managed through Cognitive Behavioral Therapy (CBT) protocols [55, 56]. This study suggests that CBT targeting cognitive biases and depressive symptoms could be beneficial in reducing the risk of psychosis in adolescents who have a history of childhood trauma, particularly within school-based health care programs for adolescents.

The interpretation of our results should take into consideration several limitations. Firstly, our sample was restricted to adolescents, potentially limiting the generalizability of our findings to the broader population. Secondly, our study adopted a cross-sectional design and utilized structural equation modeling to investigate the interplay between childhood trauma, depression, neuroticism, and cognitive biases in predicting PLEs. Although these findings provide valuable insights into the theoretical relationships among the studied markers, future research would greatly benefit from employing a longitudinal or follow-up design to further explore these relationships and establish empirical evidence of causality. Additionally, we did not account for the influence of a broader range of mood symptoms and cognitive biases, such as mania, negative symptoms, or jumping to conclusions, which are known predictors of PLEs [10, 57]. Our methods should be regarded as a general estimation of these markers, similar to those used in previous studies [45]. In the path analysis, we excluded several items with low loading from our structural equation modeling, which is an important consideration when interpreting the results. Furthermore, due to the cultural reluctance among Chinese people to discuss topics related to sexuality, questionnaires on sexual abuse were not incorporated into this study. However, sexual abuse, a pivotal aspect of childhood trauma and identified as a risk factor for the development of PLEs in prior researches [45, 58], should be given consideration in future investigative endeavors. Moreover, while this study measured PLEs using a self-report scale, future studies could benefit from incorporating clinical interviews to identify high-risk individuals and obtain more detailed clinical information. And various psychosocial factors, including sleep problems, suicidality, cannabis use, and perceived stress, have been established as significant contributors to PLEs [59–62]. It is crucial for future research to delve deeper into additional psychosocial factors to gain a more comprehensive understanding of PLEs.

In conclusion, our results suggest that neuroticism, cognitive biases, and depression play crucial roles in the relationship between childhood trauma and PLEs. These findings indicate a clear interactive effect of these markers on PLEs among adolescents.

## Electronic supplementary material

Below is the link to the electronic supplementary material.

Supplementary Material 1

## Data Availability

No datasets were generated or analysed during the current study.
