# Supplementary material for: Predicting psychotic-like experiences among adolescents: the interplay of childhood trauma, cognitive biases, neuroticism, and depression
Source: Child Adolesc Psychiatry Ment Health. 2025 Mar 14;19:20. doi: 10.1186/s13034-025-00878-5 (PMC11909816; doi:10.1186/s13034-025-00878-5)
Supplement: Supplementary file 1 — Supplementary Material 1 [file 13034_2025_878_MOESM1_ESM.docx]

Table S1. The results of confirmatory factor analyses of measures (n= 4087)

| Model | RMSEA | 95% CI | SRMR | CFI | GFI |
| --- | --- | --- | --- | --- | --- |
| CAPE – 3 factors | 0.055 | 0.052-0.058 | 0.033 | 0.952 | 0.961 |
| PHQ – 1 factor | 0.056 | 0.051-0.061 | 0.023 | 0.980 | 0.981 |
| DACOBS – 7 factors | 0.046 | 0.045-0.047 | 0.045 | 0.908 | 0.930 |
| CTQ – 8 factors | 0.055 | 0.053-0.057 | 0.062 | 0.921 | 0.946 |
| Neuroticism – 6 factors | 0.053 | 0.047-0.059 | 0.029 | 0.907 | 0.987 |

CAPE, Community Assessment of Psychic Experiences; PHQ, Patient Health Questionnaire; DACOBS, Davos Assessment of Cognitive Biases; CTQ, Childhood Trauma Questionnaire; Neuroticism, Neuroticism subscale of the Neuroticism-Extraversion-Openness Personality Inventory; RMSEA, The Root Mean Square Error of Approximation; SMRM, The Standardized Root Mean Square Residual; CFI, Confirmatory Fit Index; GFI, Goodness-of-Fit.
